# A Genome-Wide Detection of Copy Number Variations Using SNP Genotyping Arrays in Braque Français Type Pyrénées Dogs

**DOI:** 10.3390/ani9030077

**Published:** 2019-03-01

**Authors:** Rosalia Di Gerlando, Salvatore Mastrangelo, Maria Teresa Sardina, Marco Ragatzu, Andrea Spaterna, Baldassare Portolano, Filippo Biscarini, Roberta Ciampolini

**Affiliations:** 1 Dipartimento Scienze Agrarie, Alimentari e Forestali, University of Palermo, 90128 Palermo, Italy; salvatore.mastrangelo@unipa.it (S.M.); mariateresa.sardina@unipa.it (M.T.S.); baldassare.portolano@unipa.it (B.P.); 2 Club Italiano Braque Français Type Pyrénées, 58011 Capalbio, Italy; marcoragatzu@gmail.com; 3 Scuola di Scienze Mediche Veterinarie, University of Camerino, 62024 Matelica, Italy; andrea.spaterna@unicam.it; 4 Centro Interuniversitario di Ricerca e di Consulenza sulla Genetica e la Clinica del Cane, 62024 Matelica, Italy; roberta.ciampolini@unipi.it; 5 Consiglio Nazionale delle Ricerche-Istituto di Biologia e Biotecnologia Agraria, 20133 Milano, Italy; filippo.biscarini@ibba.cnr.it; 6 Dipartimento di Scienze Veterinarie, University of Pisa, 56100 Pisa, Italy

**Keywords:** copy number variation (CNV), canine high-density SNP array, Braque Français type Pyrénées dogs

## Abstract

**Simple Summary:**

Copy number variations (CNVs) are important sources of variation in mammalian species. In this study, we used a single nucleotide polymorphisms (SNP) array to detect CNVs in Braque Français, type Pyrénées dogs (BRA). Results overlapped moderately in comparison with previous studies on CNVs in dogs, leading to the identification of 16 novel CNVRs. Several genes were annotated in the CNV regions (CNVRs) detected, some of which related to muscle structure development. This breed is known to be excellent upland game birds dogs. The selection for such hunting behavior could have driven the presence of these genes into the CNVRs. Copy number variations may be of interest to study associations between genomic and phenotypic variation.

**Abstract:**

Copy number variants (CNVs) are an important source of genetic variation complementary to single nucleotide polymorphisms (SNPs). Only few studies have been conducted in dogs on CNVs derived from high-density SNP array data, and many canine breeds still remain uncharacterized, e.g., the Braque Français, type Pyrénées breed (BRA). Therefore, in an effort to more comprehensively investigate the canine genome for CNVs, we used a high-density SNP array (170 K) to discover CNVs in BRA. The CNV regions (CNVRs) were identified through the merging of two different CNVRs datasets, obtained separately from SNP data using the PennCNV and SVS software. A total of 45 stringent CNVRs, ranging from 3.5 kb to 458,716 kb in length were detected in 26 dog samples. Results overlapped moderately in comparison with previous studies on CNVs in dogs, leading to the identification of 16 novel CNVRs. A total of 159 genes were annotated in the CNVRs detected with stringent quality criteria in particular high classification stringency and false discovery rate correction. The gene ontology enrichment analysis provided information on biological processes and cellular components related to muscle structure development and muscle cell differentiation. Considering that BRA is a breed used for speed in hunting and retrieval, for the ability to find feathered game, and for pointing, we can hypothesize that selection for such hunting behavior could have driven, at least in part, the presence of these genes into the CNVRs.

## 1. Introduction

**Figure 1:**
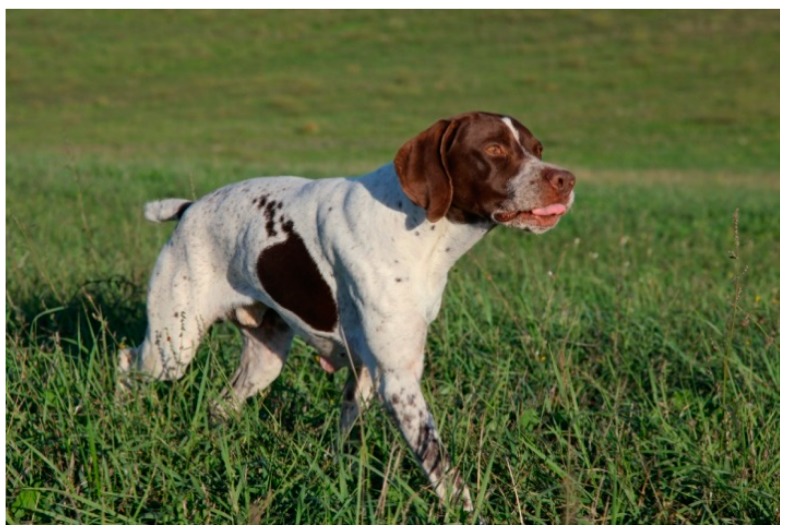
Example of Braque Français, type Pyrénées breed.

The domestication of modern dogs started approximately 15,000 years ago [1] and has resulted in an extraordinary amount of variation in canine forms and functions, through the creation of divergent breeds. Dogs, with their great biological diversity, are very suitable model animals to address studies of population genetics, phenotypic variation and diseases, many of which are similar to human diseases [2]. With the completion of the assembly of the canine genome, high-throughput sequencing and genotyping technologies offered a great potential to increase our understanding of the genomic basis of canid variation. Single nucleotide polymorphism (SNP) data have been used to investigate patterns of genetic variation within and between breeds, examine relatedness among breeds, and identify selection signatures [3]. In addition to SNPs, there are other components of the canine genomic variation, such as copy number variations (CNVs). These are important sources of genetic and phenotypic variation in mammalian species. Copy number variations have been identified in the human genome [4] and in the genomes of several livestock species, e.g., cattle [5,6], sheep [7], and pigs [8]. A number of studies have been conducted also in dogs [2,9,10,11], and some have shown the usefulness of the CanineHD array in CNV detection [12]. However, several canine breeds still remain uncharacterized, since their CNV distribution in the genome has not yet been analyzed, as for example the Braque Français, type Pyrénées breed (BRA). Individuals of BRA are a smart, agile, and friendly dogs originally developed for tracking, hunting, and retrieving feathered game. Very little is known with certainty about the origin of BRA, as this breed was developed before written records of dog breeding began to be kept [13,14]. This breed is known to be excellent upland game bird dogs which are especially skilled at hunting woodcocks (Figure 1). In an effort to more comprehensively investigate the canine genome for CNVs, we used a high-density SNP array (170 K) to detect known and novel CNVs in Braque Français, type Pyrénées dogs.

## 2. Materials and Methods

### 2.1. Sampling and Genotyping

Blood samples were collected from 48 individuals (27 females, 21 males). Genotyping was performed with the Illumina CanineHD BeadChip. The genomic positions of the SNPs on the chromosomes were obtained from the CanFam3.1 genome sequence assembly. After excluding SNPs which were unmapped or mapped to sex chromosomes, a total of 167,183 markers were used. The samples were provided by “ENCI” (Ente Nazionale Cinofilia Italiana), the institution that officially manages data for all dog breeds registered in Italy. Therefore, no ethical approval was required.

### 2.2. CNV and CNVR Detection

Copy number variations were detected from High Density SNP data using the algorithms implemented by PennCNV and in the Copy Number Analysis Module (CNAM) of the SVS 8.7.0 software (Golden Helix, Bozeman, MT, USA). These algorithms have been developed for CNV analysis from SNP array data [6,15,16]. The PennCNV incorporates multiple factors, including the log R ratio (LRR), B Allele Frequency (BAF), the marker distance, and the population frequency of the B allele (PFB). The LRR and BAF values for each SNP were obtained with the GenomeStudio 2.0 software (Illumina Inc., San Diego, CA, USA). The PFB file was calculated with PennCNV based on the BAF value for each marker in each individual. PennCNV integrates a computational approach by applying a regression model to the GC content to reduce waviness. Copy number variations were also detected using the Hidden Marckov Model parameter file. The quality of the final dataset was assessed with the criteria: a logR ratio standard deviation (LRR_SD) < 0.30, BAF drift < 0.01, and waviness factor (WF value) >0.05 or <−0.05 for each sample. To reduce the possible false CNV calls, we also considered only those CNVs that contained three or more consecutive SNPs. The Copy Number Analysis Module (CNAM) implemented in the SVS 8.7.0 software was also used for CNVs identification. The following options in CNAM were chosen: univariate outlier removal; maximum number of 100 segments per 10,000 markers; minimum markers per segment 3; 2000 permutations per pair with a *p* value cutoff of 0.005. Individuals that had −0.05 > WF > 0.05 were also excluded”. For more details on the quality criteria used to detect CNVs with the two algorithms, see Di Gerlando et al. [6]. Copy number variation regions (CNVRs) were determined by aggregating the overlapping CNVs identified across all samples [4]. Overlapping regions were calculated using BEDTools [17] and considering only those presented in at least two samples. The CNVRs obtained from both PennCNV and SVS were determined by intersecting the datasets and inferring the overlapping CNVRs using the approach described by Wain et al. [18], which identifies CNVRs that fully overlap.

### 2.3. Gene Contents and Functional Annotation

The gene content of CNVRs was assessed based on CanFam 3.1 in the Genome Data Viewer genome browser from the US National Center for Biotechnology Information (NCBI) database (https://www.ncbi.nlm.nih.gov/genome/gdv/browser/?context=genome&acc=GCF_000002285.3). The g:GOSt tool from g:Profiler (https://biit.cs.ut.ee/gprofiler/index.cgi) was used for Gene Ontology (GO), while pathway analysis was based on the Kyoto Encyclopedia of Gene and Genomes (KEGG) database of annotations. Results were corrected for multiple testing using g:False Discovery Rate (https://biit.cs.ut.ee/gprofiler/gost). 

## 3. Results and Discussion

### 3.1. CNV and CNVR Detection

**Figure 2:**
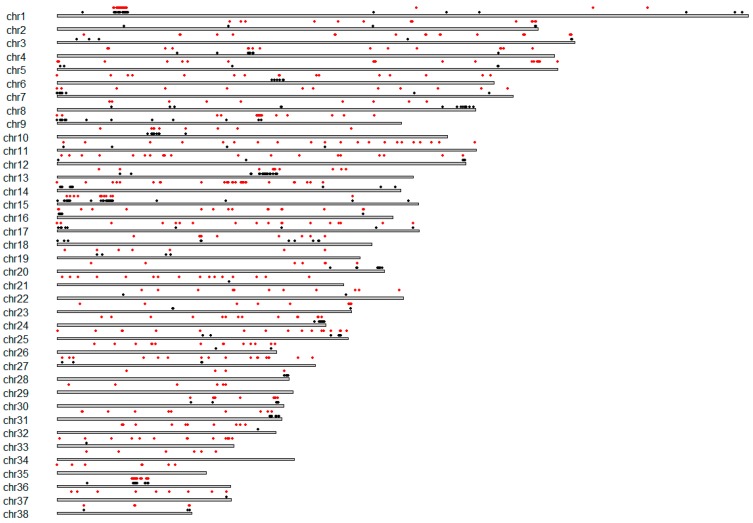
Genomic distribution of copy number variations (CNVs) in Braque Français, type Pyrénées breed. The points in black showed the CNVs identified with PennCNV, whereas in red are reported the CNV identified with Copy Number Analysis Module-SVS.

After stringent quality filters, genome-wide CNV analysis was conducted on the remaining 26 BRA samples. In this way we filtered out most of the potentially noisy data that would have reduced the reliability of the called CNVs [19]. However, the final number of BRA samples was higher compared with the number used in a previous study of CNV discovery in several dog breeds [12]. Using PennCNV, a total of 1047 CNVs (Supplementary Materials Table S1: CNVs_Penn, Figure 2) were detected, with an average length of 107.123 kb and average number of 40.3 CNVs per sample. The CNAM univariate segmentation of SVS identified 1638 (Supplementary Table S1: CNVs_SVS, Figure 2) with an average length of 110.41 kb and the average number of 63 CNVs per sample. The frequency of CNVs in the population for PennCNV ranged from 0.96 × 10^−3^ and 0.015, and for CNAM-SVS ranged from 0.61× 10^−3^ and 0.013. The ratio between duplications and deletions was of 1:4 and 1:136 for PennCNV and CNAM-SVS, respectively. By aggregating the overlapping CNVs from PennCNV, a total of 181 CNVRs (Supplementary Materials Table S1: CNVRs_Penn) were identified. The total autosomal CNVRs coverage was 15.75 Mb, which corresponds to 0.7% of the canine autosomal genome. The CNVs identified with SVS were aggregated into 280 CNVRs (Supplementary Table S1: CNVR_SVS), covering 24.11 Mb, which corresponds to 1.1% of the canine autosomal genome. The differences in the numbers of CNVs and CNVRs detected by the two algorithms were probably attributable to the identification of longer CNVs with the univariate SVS approach [19].

Intersecting the two CNVR datasets (from PennCNV and SVS software) a total of 45 CNVRs (Supplementary Materials Table S1: CNVR_Penn_vs_SVS), distributed on the 38 autosomes, were obtained. The CNVRs from PennCNV that did not fully overlap with SVS results were excluded, and vice versa. This strategy, where the final list of stringent CNVRs was obtained from both algorithms, has been recommended previously in order to reduce the false discovery rate [20]. Among the stringent CNVRs, 42 CNVRs were defined as deletions, and only three as loss/gain, meaning that both deletions and duplications were observed. The length of these CNVRs ranged from 3.5 kb to 458.716 kb with an average size of 87.92 kb. In particular, 49% CNVRs were smaller than 50 kb, 18% were in the range of 50–100 kb, and 33% were larger than 100 kb. Different values were reported by Molin et al. [12] who, by using the CanineHD array in 26 dog breeds, found the highest CNVRs proportion (64%) in the size-range 100 kb–1 Mb.

### 3.2. Comparison with Published CNVRs

**Figure 3:**
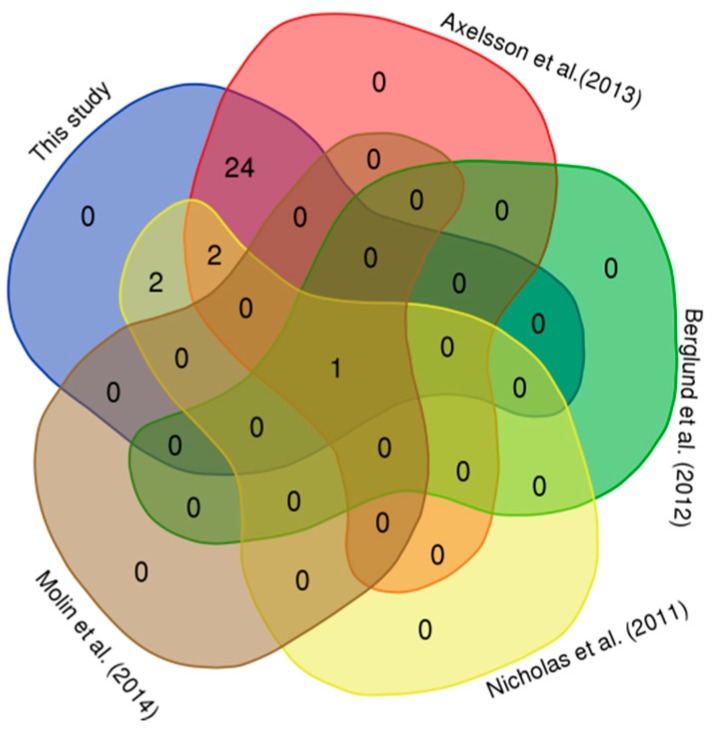
Comparison between identified CNVs in this and other studies.

For a correct interpretation, our results were compared with canine CNVRs from the available literature. To this end, we converted the 45 CNVRs’ map positions to the previous version of canine genome (CamFam 2.0). When comparing CNVRs in BRA to the previously identified CNVRs in dogs, we found almost 64% (29/45) of overlapping regions (Table 1 and Figure 3). The highest number of overlapping CNVRs (27/45) was found in the study of Axelsson et al. [21], in which CNV detection was performed using whole-genome re-sequencing. Only one CNVR, CNVR36 on chromosome 27, was detected in all previous considered studies [10,11,12,21]. Five CNVRs overlapped with those reported by Nicholas et al. [10]. Our results revealed 16 novel CNVRs: since the present study had been conducted on a dog breed never analyzed for CNVs previously, the possibility of identifying new and breed-specific CNVRs was high. The presence of breed-specific CNVRs have been also described in other studies on dogs [11,12]. These single-breed CNVRs are of particular interest if they are fixed or with an increased frequency in the breed, since they could be involved in breed specific characteristics [12]. Unfortunately, in our study, as well as in other previous works [11,12], no fixed single breed CNVRs were detected, and therefore these are most often not involved in breed specific characteristics. However, considering the numerous examples on the involvement of CNVs in shaping the phenotype in dogs [22], these results emphasize the necessity to discover novel CNVs in uncharacterized breeds. This is especially true for the canine species, where the study of CNV has received less attention compared to livestock species [5,6,7,8].

### 3.3. CNVR Gene Content and Functional Annotations

From our final list, 37 CNVRs overlap with 159 annotated genes (Supplementary Materials Table S1: Genes). Some of these genes are associated with well-known phenotypes in dogs. The *Sox8* gene, such as the *Sox8* gene, which is involved in sex determination [23]. The *SLC38A2* gene has been described to be potentially involved in hypoxia adaptation, from a study on high-altitude adaptation in Tibetan mastiffs [24]. The *MYOG* gene is an important myogenic regulatory factor necessary for myocyte differentiation and the development of functional skeletal muscle [25,26]. In a dog study on chronic exposure to stress, Luo et al. [27] indicated that the *ADORA* gene might be important in explaining the stress-tolerance variation. Kyoto Encyclopedia of Gene and Genomes (KEGG) annotations showed that endocrine resistance, Notch signaling pathway, and the Wnt signal pathway were significantly enriched. The detected genes encompass a wide spectrum of biological processes and cellular components. The most significant biological processes were muscle structure development (GO:0061061), muscle cell differentiation (GO:0042692), and striated muscle cell differentiation (GO:0051146). The significant cellular component was filamentous actin (GO:0031941). These are interesting results considering that BRA is a dog breed used for tracking, hunting, pointing and retrieving feathered game [14]. These are strong dogs, adequately muscled but without heaviness. We can hypothesize that selection for such hunting behavior, for which particular anatomical features are required, could have shaped, at least in part, the genetic background of this breed and, consequently, the frequency/presence of the detected CNVRs in these genes.

## 4. Conclusions

At present, limited knowledge is available on CNVs detected from HD array in dogs. This is the first CNV study in the Braque Français, type Pyrénées dog breed. The use of the CanineHD SNP array data and of two different algorithms for the identification of CNVs makes our results very robust and with low probability of false positives. Our results emphasize the necessity to discover novel CNVs in uncharacterized breeds. Additionally, as high density SNP array data are becoming increasingly available, CNVs combined with SNPs may be of interest to study associations between genomic and phenotypic variation.

## Figures and Tables

**Table 1:** List of copy number variation regions (CNVRs) detected in this study that overlapped with those reported in the literature. The symbol * shows the overlapping between studies.

CNVRs	Chromosomes	Start Position (bp)	End Position (bp)	Nicholas et al. (2011)	Berglund et al. (2012)	Axelsson et al. (2013)	Molin et al. (2014)
1	chr1	11,200,412	11,215,077			*	
4	chr4	33,991,898	34,067,769			*	
5	chr6	39,482,383	39,941,098			*	
6	chr6	40,137,436	40,261,501			*	
9	chr7	790,325	836,217			*	
10	chr7	846,445	871,741			*	
12	chr9	615,993	795,784			*	
13	chr9	660,066	909,141			*	
14	chr9	916,372	997,748			*	
17	chr10	16,943,758	1,705,5639			*	
18	chr10	17,172,641	1,728,4700			*	
20	chr10	18,100,399	18,220,792	*		*	
25	chr14	853,721	1,000,647			*	
26	chr15	8,373,335	8,384,052			*	
27	chr16	383,954	442,252			*	
28	chr17	749,155	818,590			*	
29	chr17	39,880,141	3,990,5486			*	
30	chr18	25,644,344	25,740,090			*	
32	chr23	52,156,177	5,215,9756	*		*	
33	chr25	48,611,803	48,662,322			*	
34	chr25	50,314,369	50,482,865			*	
35	chr27	2,869,179	2,886,074			*	
36	chr27	25,695,551	2,581,0881	*	*	*	*
37	chr28	40,299,547	40,325,378			*	
38	chr28	40,715,077	40,730,141			*	
40	chr30	39,280,736	39,318,243			*	
42	chr36	13,722,385	13,742,102	*			
43	chr37	30,065,232	30,112,232			*	
44	chr38	4,831,886	4,885,582	*			

## Supplementary Materials

The following are available online at https://www.mdpi.com/2076-2615/9/3/77/s1. Table S1: List of CNVs, CNVRs and annotated genes identified in Braque Français, type Pyrénées dog. CNVs and CNVRs identified with PennCNV software (CNVs_Penn and CNVRs_Penn, respectively), CNVs and CNVRs identified with SVS software (CNVs_SVS and CNVRs_SVS, respectively), common CNVRs obtained from PennCNV and CNAM-SVS (CNVR_Penn_vs_SVS), and Genes identified in CNVRs (Genes).
